# Homologous recombination deficiency (HRD) testing on cell-free tumor DNA from peritoneal fluid

**DOI:** 10.1186/s12943-023-01864-1

**Published:** 2023-11-06

**Authors:** Cyril Roussel-Simonin, Felix Blanc-Durand, Roseline Tang, Damien Vasseur, Audrey Le Formal, Laure Chardin, Elisa Yaniz, Sébastien Gouy, Amandine Maulard, Stéphanie Scherier, Claire Sanson, Ludovic Lacroix, Sophie Cotteret, Lea Mauny, François Zaccarini, Etienne Rouleau, Alexandra Leary

**Affiliations:** 1 Drug Development Department (DITEP), Gustave-Roussy Cancer Campus, Villejuif. Sorbonne Université, Paris, France; 2 Departement of Medecine, Gustave-Roussy Cancer Campus, INSERM U981, Université Paris-Saclay, Villejuif, France; 3 Université Paris-Saclay, Gustave-Roussy Cancer Campus, Inserm U981, Villejuif, France; 4 Cancer Genetics Laboratory, Medical Biology and Pathology Department, Gustave-Roussy Cancer Campus, Villejuif, France; 5 Department of Pathology and Medical Biology, Gustave-Roussy Cancer Campus, Villejuif, France; 6 Department of Gynecologic Surgery, Gustave-Roussy Cancer Campus, Villejuif, France

**Keywords:** ctDNA, Ovarian cancer, shallowWGS, HRD, Ascites

## Abstract

**Background:**

Knowing the homologous recombination deficiency (HRD) status in advanced epithelial ovarian cancer (EOC) is vital for patient management. HRD is determined by BRCA1/BRCA2 pathogenic variants or genomic instability. However, tumor DNA analysis is inconclusive in 15–19% of cases. Peritoneal fluid, available in > 95% of advanced EOC cases, could serve as an alternative source of cell-free tumor DNA (cftDNA) for HRD testing. Limited data show the feasibility of cancer panel gene testing on ascites cfDNA but no study, to date, has investigated HRD testing.

**Methods:**

We collected ascites/peritoneal washings from 53 EOC patients (19 from retrospective cohort and 34 from prospective cohort) and performed a Cancer Gene Panel (CGP) using NGS for TP53/HR genes and shallow Whole Genome Sequencing (sWGS) for genomic instability on cfDNA.

**Results:**

cfDNA was detectable in 49 out of 53 patients (92.5%), including those with limited peritoneal fluid. Median cfDNA was 3700 ng/ml, with a turnaround time of 21 days. TP53 pathogenic variants were detected in 86% (42/49) of patients, all with HGSOC. BRCA1 and BRCA2 pathogenic variants were found in 14% (7/49) and 10% (5/49) of cases, respectively. Peritoneal cftDNA showed high sensitivity (97%), specificity (83%), and concordance (95%) with tumor-based TP53 variant detection. NGS CGP on cftDNA identified BRCA2 pathogenic variants in one case where tumor-based testing failed. sWGS on cftDNA provided informative results even when tumor-based genomic instability testing failed.

**Conclusion:**

Profiling cftDNA from peritoneal fluid is feasible, providing a significant amount of tumor DNA. This fast and reliable approach enables HRD testing, including BRCA1/2 mutations and genomic instability assessment. HRD testing on cfDNA from peritoneal fluid should be offered to all primary laparoscopy patients.

**Supplementary Information:**

The online version contains supplementary material available at 10.1186/s12943-023-01864-1.

## Introduction

Ovarian cancer is the most lethal gynecological cancer worldwide accounting for 250 000 cases and 180 000 deaths annually. High-grade serous ovarian cancer (HGSOC) is the most common and aggressive subtype of ovarian cancer with a 5 year-overall survival of 30%, essentially due to the advanced stage at the time of diagnosis as most EOC are diagnosed at stage III or IV (70%) [1].

Approximatively 50% of HGSOC harbor homologous recombination deficiency (HRD) with high levels of genomic instability, either due to a germline or somatic *BRCA1/2 pathogenic variant* or to another unknown mechanism [2, 3]. Poly (adenosine diphosphate–ribose) polymerase inhibitors (PARPi) block the repair of single strand breaks thus generating double-strand breaks that cannot be repaired in tumors that are HRD [4]. Large phase III randomized clinical trials evaluating the benefit of PARPi as 1^st^ line maintenance treatment for advanced (stade III/IV FIGO) high grade OC have now clearly established that HRD status determined by *BRCA1/2* pathogenic variant and genomic instability testing predicts magnitude of benefit from this class of agents [4–8]. It is today essential that every patient with newly diagnosed high grade ovarian cancer is offered testing for *BRCA1*/*2* gene pathogenic variant and genomic instability. Unfortunately HRD testing on formalin-fixed, paraffin-embedded (FFPE) tumor samples yields non-contributive results in 15% to 19% of patients due to low tumor cellularity or poor quality DNA [5–7]. This can be particularly problematic in patients who are not candidate for primary debulking surgery where the only tumor sample may be a small biopsy obtained at diagnostic laparoscopy, or necrotic samples from interval debulking surgery post-neoadjuvant chemotherapy. More than half of women with stage III/IV high grade OC exhibit clinically evident malignant ascites at diagnosis, in addition a further subset present smaller amounts of cytologically confirmed malignant peritoneal free fluid at surgical exploration [9]. We recently demonstrated that 98% of patient with stage III/IV HGOC have at least a small amount of clinically visible peritoneal fluid (ascites) at diagnostic laparoscopy or laparotomy (submitted). This peritoneal fluid known to contain tumor cells could provide an alternative liquid biopsy sample for HRD tumor testing.

Cell-free tumor DNA (cftDNA) has been studied for the last 30 years and can be detected in various body fluids including blood, urine, pleural fluid and ascites [10]. It is now commonly used [11] in some cancers like *EGFR* mutated non-small cell lung cancer [12]. However, cfDNA in ascites (acfDNA) has been less studied. Some have shown that acfDNA can be detected in gastric [13] or colon cancer [14]. Han and colleagues [15] identified nine somatic pathogenic variants in matched tumor tissue and ascites from 10 OC patients. Another study confirmed high concentrations of cftDNA in ascites from 18 OC patients and intriguingly, showed that cftDNA yielded higher variant allele frequency in ascites compared to than DNA extracted from tumor cells [16]. These results suggest that cftDNA from ascites in patients with advanced EOC can provide a surrogate liquid biopsy to characterize the genomic landscape of ovarian cancer.

In this study, we aimed to evaluate the feasibility and clinical usefulness/performance of HRD testing on cftDNA from peritoneal fluid in newly diagnosed advanced ovarian cancer patients. A Cancer Gene Panel (CGP) including *TP53* (as a control to confirm detection of tumor DNA) as well as *BRCA1/2* and other HR-related genes, was analysed by Next Generation Sequencing (NGS) on matched tumor DNA from FFPE tissue samples and cftDNA from ascites from 53 patients presenting with advanced OC at Gustave Roussy’s cancer center. In addition, we performed genomic instability testing on FFPE tumors and cftDNA from peritoneal fluid samples.

## Material & methods

### Identification of patient and collection of samples

All patients provided written informed consent authorizing the use of biological samples obtained during their routine diagnosis and treatment as part of the prospective academic research study OvBIOMARK (NCT03010124).

In a first pilot phase, we retrospectively analyzed ascites collected between 2017 and 2021 during primary or secondary laparoscopy, from 19 patients with confirmed EOC. At that time, 15 mL of freshly collected ascites were double centrifuged at 1000 g and 14 000 g within an hour of collection and the supernatant was frozen at -80 °C.

We then prospectively collected 15–20 ml of peritoneal fluid samples between January 2022 and August 2022 from patients with suspected or confirmed epithelial ovarian cancer during primary or secondary laparoscopy/laparotomy. Those subsequently confirmed as non-EOC primaries (such as gastro-intestinal tumor or endometrial carcinoma) were excluded from further analysis. In addition, we collected 7 ascites from OC patients who required paracentesis for symptom control in the relapsed setting. Finally for 7 patients who did not present any visible peritoneal fluid at laparoscopic exploration, peritoneal washings were collected (referred to as indirect ascites).

In this second prospective phase, 20 ml of ascites or peritoneal washings were collected into 2 cell-free DNA collection tube of 10 ml each (PAXgene®). Peritoneal fluid (ascites) or washings (indirect ascites) were centrifuged within 4 h after the collection at 1500 g for 10 min. The supernatant was then centrifuged a second time at 20 000 g for 10 min and the supernatant was frozen at -80 °C.

### DNA extraction from ascites and CGP

cfDNA was extracted from 1 to 4 ml of the centrifuged fluid using the QIASymphony Circulating DNA Kit (Qiagen) following manufacturer's instruction, or Maxwell® RSC ccfDNA Plasma Kit (Promega) following manufacturer's instruction. The quality control of cfDNA were analyzed with the Cell-free DNA ScreenTape assay (Agilent).

The NGS CGP on matched FFPE tumor sample and cfDNA for the first 19 patients covered 65 genes including *TP53*, *BRCA1/2* and other HR-related genes. In the prospective part of the study (*N* = 34 pts), the panel was extended to 109 genes. The details regarding the 65 and 109 panel gene can be found in Supp Data.

A sample was considered as containing Cell free DNA if a mononucleosomal spike was detected at 160-bp and further spikes every 160-bp between 160 to 700 bp. The presence of these replicate spikes was considered a marker of cfDNA quality. Samples were considered as containing poor quality cfDNA for NGS if the first spike at 160-bp was not visible and as poor quality for sWGS as detailed in the publication by Eeckhoutte, A. et al. [17]*.*

### Genomic instability testing

Every patient included in our cohort had a genomic instability test (MyChoice Myriad®) conducted on DNA from formalin-fixed paraffin embedded (FFPE) tumor tissue specimen. This assay allows the determination of a Genomic Instability Score (GIS) which is an algorithmic measurement of Loss of Heterozygosity (LOH), Telomeric Allelic Imbalance (TAI), and Large-scale State Transitions (LST). Sample is considered HRD if GIS is ≥ 42.

The MyChoice Myriad® assay cannot be performed on cfDNA, thus we evaluated genomic instability in cftDNA samples using Shallow Whole Genome Sequencing (sWGS). This approach represents an attractive alternative as it requires low DNA input, it is fast and cost effective [17]. Genomic instability testing using sWGS used the shHRD algorithm. This algorithm is based on the number of large-scale genomic alteration (LGA) which is defined as intra-chromosome arm CNA breaks with adjacent segments ≥ 10 Mb. It is the reflection of LST in Myriad® HRD scar assay. A given sample is considered HRD if LGA is ≥ 20 and is considered HR proficient if LGA is < 15. Samples with LGA between 15 and 19 are considered “borderline” [17].

## Results

### Patient characteristics

**Fig. 1:**
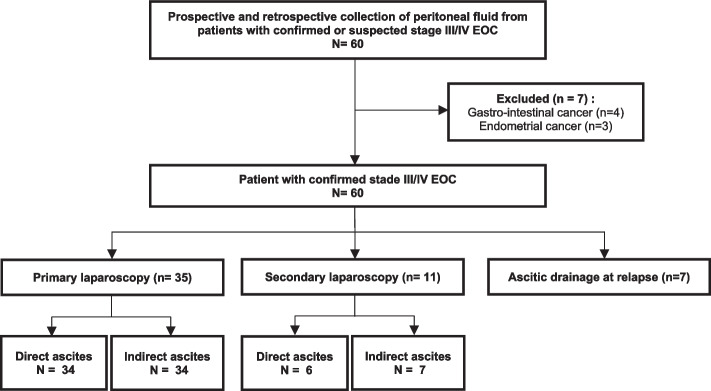
Flow Chart

A total of 53 patients were included in our study (Fig. 1). 19 patients from the retrospective pilot study (ascites at 1° laparoscopy (*N* = 18) or interval 2° surgery/laparoscopy (*N* = 1)) and 34 from the prospective study (ascites at 1° laparoscopy (*N* = 17), ascites at 2° laparoscopy (*N* = 10) or therapeutic paracentesis *N* = 7). The high rate of ascitic sample collection at interval surgical exploration in our series is attributable to the fact that some patients were referred to our center for debulking surgery after 3–4 cycles of neoadjuvant chemotherapy.

Direct ascites was collected for 34/35 of samples obtained at 1° laparoscopy, for 5/11 at 2° laparoscopy and 7/7 at paracentesis. Among patients with visible ascites at laparoscopy (*N* = 39), ascitic volume was 20-100 ml in 18% (7/39), 100-500 ml in 8% (3/39), 500-1000 ml in 28% (11/39) and > 1000 ml in 46% (18/39). For patients without visible ascites (at 1°, *N* = 1 or 2° laparoscopy, *N* = 6), indirect ascites was obtained by peritoneal washings with saline.

Median age was 65 years old (range: 42 – 86) and most patients had FIGO stage III/IV (98%), and high grade ovarian cancer (92%, 49/53) other histologies included low grade ovarian cancer (*N* = 4). Most ascites and tumor sample were collected at primary laparoscopy (66%) (Table 1).

**Table 1:** Characteristics of patient at baseline

	**Total (*n* = 53)**
**Age (years)**	
Means (SD)	65 (± 10)
**ECOG at sample**	
0	13 (25%)
1	33(62%)
2	7 (13%)
**Histology**	
Serous High grade	47 ( 89%)
Serous Low Grade	4 (8%)
Endometrioid	2 (4%)
**Primary Tumor Location**	
Ovary	44 (83%)
Peritoneum	5 (9%)
Fallopian Tube	4 (8%)
**International FIGO stage**	
II	1 (2%)
III	32 (60%)
IV	20 (38%)
**Type of Sample**	
Laparoscopy	46 (87%)
Therapeutic paracentesis	7 (13%)
**Status at sample**	
Primary laparoscopy	35 (66%)
Secondary laparoscopy	11 (21%)
Relapse	7 (13%)
**Amount of peritoneal fluid at sample**	
Absence	7 (13%)
< 500 cc	10 (19%)
500 to 1000 cc	12 (23%)
> 1000 cc	24 (45%)

### Contributive cftDNA detection from ascites

**Fig. 2:**
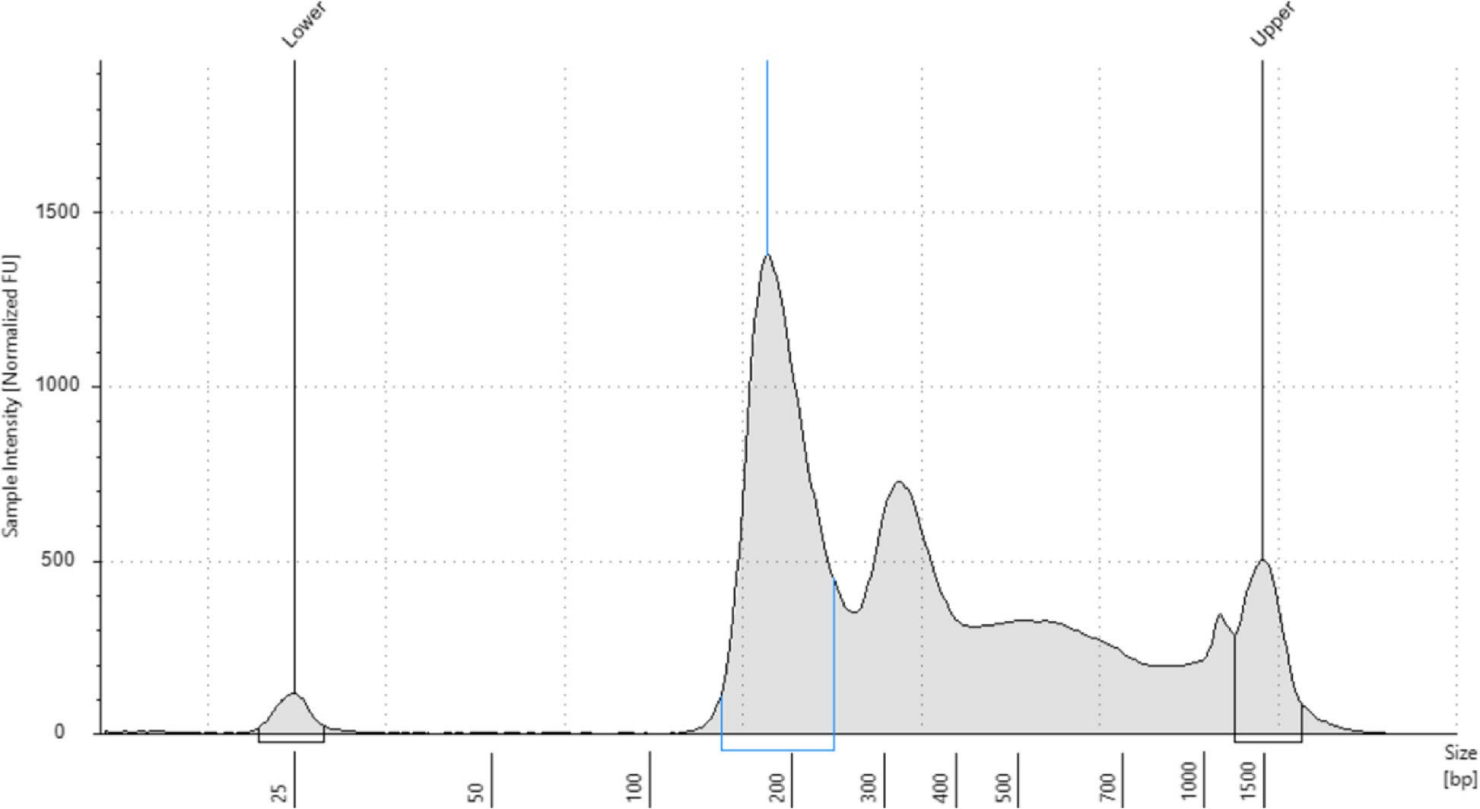
Example of patient’s electropherogram: sizing range of DNA detected in ascites

Cell-free DNA was detectable in peritoneal fluid from 49/53 patients (92,5%). Overall, DNA quality was high. Figure 2 illustrates the typical profile of cfDNA extracted from peritoneal fluids in our study featuring a single mononucleosomal peak at a mean length of 177 bp (range: 128 – 204), along with two additional peaks at around 360 and 520 bp. This showcases the high quality of cfDNA extracted from peritoneal fluids with low contamination by high molecular weight DNA (> 700-pb).

Additionnaly, DNA yield was very high with a median concentration of total cfDNA of 3700 ng/ml (range 109 – 65 000 ng/ml). The reported cfDNA concentration represents the concentration of cfDNA with a size range of less than 1000 base pairs in the extracted material (from 1 to 4 ml of ascites). For patients with peritoneal lavage the concentration was also high with a median concentration of 1310 ng/ml (range: 1000-2120 ng/ml). In comparison, cfDNA concentration extracted from plasma in patients with any solid tumor usually ranges from 5 to 1500 ng/ml [18]. When considering only direct ascites, cfDNA was detected in 100% of cases (46/46), including ascites obtained after neoadjuvant chemotherapy at 2° laparoscopy. Importantly direct ascites yielded cfDNA regardless of volume present. Among the patients with < 100 ml (*N* = 7) or 100-500 ml (*N* = 3), cfDNA was detected in all cases and median concentration of cfDNA for patient with < 500 ml ascite was 3150 ng/ml (range: 20,9 – 15 900 ng/ml). Interestingly, in patients without visible peritoneal free fluid and in whom peritoneal washings were collected, cfDNA was still detected in 42% of these cases (3/7). The characteristics of the 4 patients without detectable cfDNA are summarized in Table 2. All samples were obtained at 2° laparoscopy after a median of 3,5 cycles of neoadjuvant chemotherapy, via peritoneal washings and all patients demonstrated a good clinico-biological response to chemotherapy.

**Table 2:** Characteristics of patient with cfDNA analysis on peritoneal washings

	cfDNA- on peritoneal washing	cfDNA + on peritoneal washing
	Total (*n* = 4)	Total (*N* = 3)
**Histology – n (%)**		
Serous High Grade	3 (75)	3 (100)
Endometrioid	1 (25)	
**Type of ascites – n (%)**		
Indirect	4 (100)	3 (100)
**Status at sample – n (%)**		
Primary laparoscopy	0 (0)	1 (33,3)
Secondary laparoscopy	4 (100)	2 (66,7)
**Neoadjuvant treatment – n (%)**		
Carboplatine Taxol	3 (75)	2 (66,7)
Carboplatine and Gemcitabine	1 (25)	
**Number of cycle of chemotherapy – n (%)**		
Median (IQR)	3,5 (± 1,5)	6
**Response to chemotherapy – n (%)**		
Biological response	4 (100)	2 (66,7)
Radiographical response	4 (100)	2 (66,7)
Histological response	3 (75)	0 (0)

### Pathogenic variant detection on cftDNA from peritoneal fluid

**Fig. 3:**
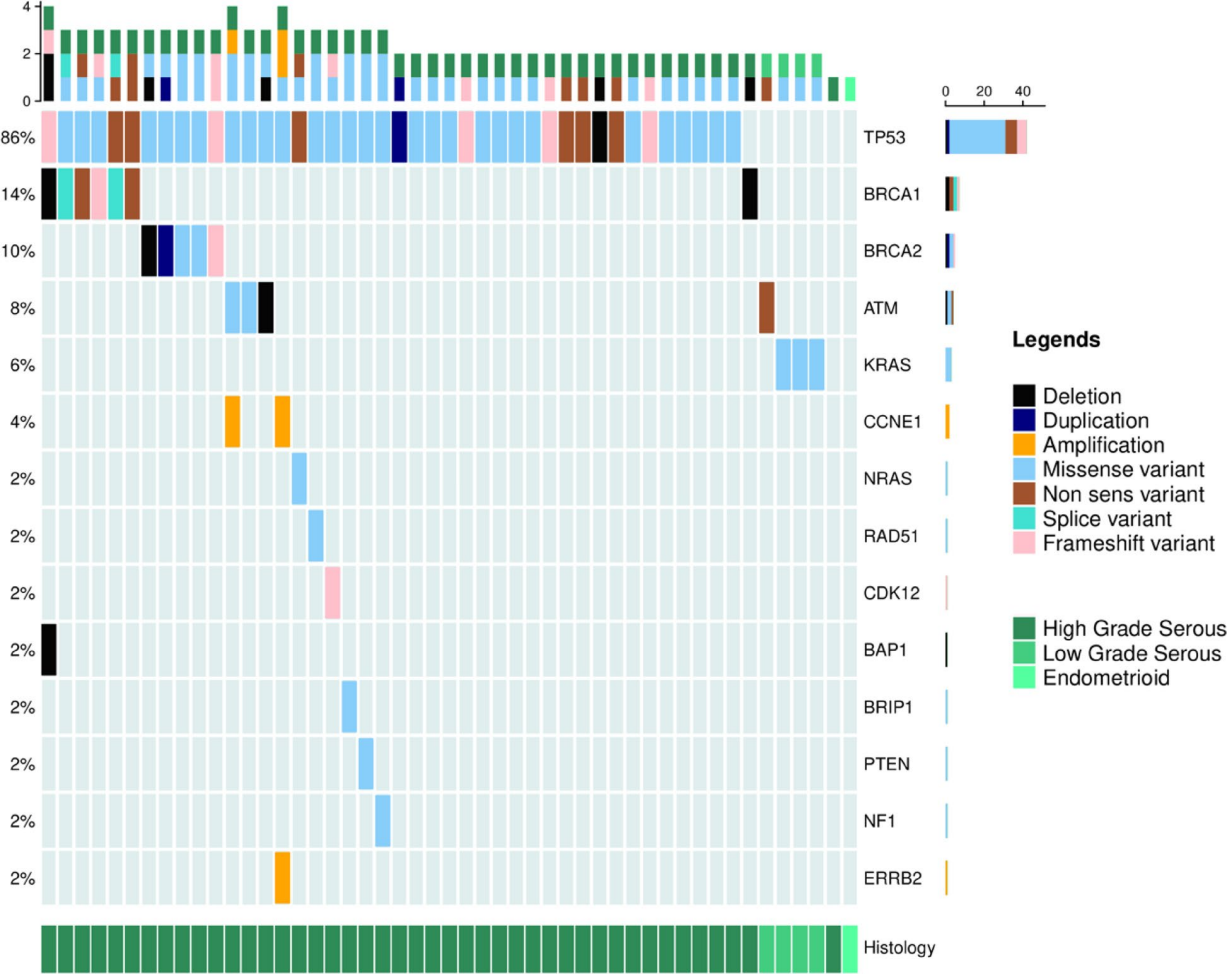
Oncoprint for CGP testing on cfDNA

Crucially, among the 49 of 53 cases with detectable cfDNA, a pathogenic variant was detected in 96% of peritoneal fluid samples (47/49), thus confirming that when cfDNA was detected it almost invariably contained tumoral DNA (cftDNA) (Fig. 3). For 1 of the 2 patients for whom no pathogenic variant was detected on ascites cftDNA (acftDNA), the NGS CGP also did not identify a pathogenic variant on tumor tissue.

The most common pathogenic variant identified in peritoneal fluid samples was *TP53* detected in 86% of 49 contributive samples (Fig. 3). Three low grade OC acftDNA harbored a *RAS* pathogenic variant and one had ATM pathogenic variant. *BRCA1* and *BRCA2* pathogenic variants were detected in respectively 14% (7/49) and 10% (5/49) of patients including one large *BRCA1* rearrangement (deletion of exon 21 to 24) detected on acftDNA, confirming the high quality of the acftDNA to detect such a quantitative event. Finally, the median testing turn-around time was only 21 days (range: 14–36) for NGS on acftDNA.

### Genomic testing on cftDNA from peritoneal fluids vs tumor samples

As our main objective was to evaluate the performance of HRD testing on peritoneal cftDNA, we focused on HGOC pts (*N* = 49) and compared the performance of cftDNA to tumor tissue based testing. NGS CGP on DNA from FFPE matching tissue samples identified a pathogenic variant in 90% (44/49) of cases. Median testing turn-around time was longer than for acftDNA at 45 days (range: 14–96). cfDNA NGS on peritoneal samples from HGOC was comparable with mutated cftDNA identified in 88% (43/49). However if the analyses were limited to cftDNA identified in direct ascites, ctDNA detection rate was higher at 98% (45/46). The patients for whom no pathogenic variant was detected on ascites cfDNA (acfDNA), the NGS CGP also did not identify a pathogenic variant on tumor tissue. For the 5 patients with a failed tumor tissue analysis, a TP53 mutation was detected in the matching ascites sample, including one patient for whom acftDNA analysis identified a *BRCA2* pathogenic variant. Together these data support that cftDNA from ascites may allow physicians to salvage non-contributive tumor analyses.

Subsequently, an evaluation of sensitivity, specificity, and concordance was conducted to compare acfDNA (cell-free DNA from ascites) with tumor testing for the detection of TP53 pathogenic variants.

This analysis was specifically focused on patients diagnosed with high-grade ovarian carcinoma who exhibited contributive results in both cfDNA and tumor tissue analyses (*N*=42, 80% of cohort).

Sensitivity and sensibility analyse were perform using the contingency table in Table 3 with GraphPad Prism version 10.0.0. Confidence interval were estimate using the The hybrid Wilson/Brown method Kappa Cohen analysis was conducted using R Studio (v 3.3.0, R Foundation for Statistical Computing, Vienna, Austria. https://www.R-project.org/) with the kappa.cohen function to evaluate concordance for this study. The concordance column in Table 3 correspond to the percentage of agreement between acfDNA and tissue analysis.

**Table 3:** Assay Positive Predictive Value, Negative Predictive Value, Specificity, Sensitivity, and Concordance of TP53 pathogenic variant on acfDNA compared With Tumor Tissue

	**AcfDNA Positive sample**	**acfDNA negative sample**	**Sensitivity,**	**Specificity,**	**Concordance,**	**Cohen K**,
			%(95%,CI)	%(95%,CI)	%	(*P* value)
**Tissue**	35	1				
**Positive**			97	83	95	0,81
**Tissue**	1	5	(86–100)	(43 – 100)		(< 0,001)
**Negative**						

The sensitivity,specificity and concordance were 97% (95% IC : 86%-100%), 83% (95% IC : 43%-100%) and 95% (K = 0,81: *P* <0,001) respectively (Table 3). One discordant patient harbored a *TP53 *pathogenic variant detected on acftDNA that was not detected on tissue DNA, potentially attributable to low cellularity and inversely one patient had TP53 pathogenic variant detected on tissue DNA but not on acftDNA.

Mean variant allele frequency (VAF) in ascites also compared favorably to tissue. The mean VAF for *TP53* pathogenic variant were 54% in acftDNA vs 45% in ttDNA. For *BRCA1* pathogenic variant the mean VAF was 67% in acftDNA and 63,3% in ttDNA and for *BRCA2* pathogenic variant it was 80% in acftDNA and 84,5% in ttDNA.

However, suprisinsigly,examination of the individual Variant Allele Frequencies (VAFs) of TP53 in tissue and ascites using Pearson correlation analysis revealed no significant correlation between these two sample types (*r* = 0.19, *p* = 0.27, see Supplementary Fig. 1). The statistical analyses were conducted using the cor.test function in R Studio (version 3.3.0, R Foundation for Statistical Computing, Vienna, Austria, https://www.R-project.org/).

### Genomic instability testing

Tumor-based genomic instability testing (Myriad MyChoice CDx) was performed on all HGOC samples as part of routine care, 3 results were pending at the time of publication. 44 patients had result available with 75% (33/44) yielding a contributive result. Among the 32 patients with successful tumor-based genomic instability testing, 16 (50%) were considered HRD + with a GIS > 42.

Genomic instability using shallow WGS (sWGS HRD) was measured on 18 acftDNA samples (including 4 with failed tumor-based GIS). All 18 patient had direct ascites sample. sWGS HRD was successful for all 18 samples, including the 4 with failed tumor testing resulting in a 100% contributive genomic instability test result and 10/18 acftDNA samples exhibited high genomic instability (LGA > 20) thus confirming the feasibility of performing genomic instability testing on cftDNA from ascites.

## Discussion

We show for the 1^st^ time the feasibility and clinical usefulness of performing HRD testing encompassing both *BRCA1/2* pathogenic variant analysis as well as genomic instability testing on cftDNA from peritoneal fluid obtained from patients with newly diagnosed advanced ovarian cancer. These ascitic samples yielded contributive cfDNA in 92,5% of cases overall, and in 100% of cases at primary surgical exploration. Importantly when cfDNA was detected, it was confirmed as tumoral in 96% of cases. acftDNA quality and quantity was excellent and median turn-around testing time was very acceptable at 21 days, shorter than for tumoral analysis. Median TP53 VAF, an accepted surrogate for tumor cellularity in OC, was superior to 50%, highlighting tumor DNA enrichment. Importantly, less than 20 ml of ascites is required and acftDNA analysis was contributive even in patients with less than 100 ml ascites. In an effort to evaluate how broadly applicable this approach may be, we previously showed that 98% of patients with newly diagnosed stage III/IV OC presented at least 100 ml of free peritoneal fluid at primary surgical exploration and would have been candidate for a liquid biopsy approach.

These data support that cfDNA from small volumes of direct or indirect ascites yields quality tumor derived DNA suitable for genomic analysis. Our next priority was to demonstrate the clinical usefulness of this liquid biopsy as a tool for HRD testing. *BRCA1/2* pathogenic variant were identified in 24% of acftDNA samples and for those with matching tumor NGS results. Regarding genomic instability testing, test failure rate was high in ttDNA from tissue samples (25%). In contrast, genomic instability testing using a sWGS approach was feasible in 100% of acftDNA samples and was able to salvage 100% of failed tumor based GIS testing. Taken together these data suggest that acftDNA analysis from peritoneal fluid provides a suitable alternative for HRD testing on tissue and could be used in place of tumor testing and can provide a rescue strategy in the event of uninformative tumor testing.

All genomic testing whether it is on acftDNA or ttDNA should ideally be conducted on treatment naïve samples from initial diagnosis. However, in the event that genomic testing was not performed or failed at primary diagnosis, our results suggest that HRD testing can also be performed on ascites collected at interval debulking after neoadjuvant chemotherapy. We even confirmed the usefulness of peritoneal washings in patients without any free fluid as these yielded detectable cftDNA in 43% of cases.

The sensitivity, specificity and concordance of acfDNA compared with tumor testing for TP53 pathogenic variant detection were 97% (95% IC: 86%-100%), 83% (95% IC: 43%-100%) and 95% (K = 0,81: *P* < 0,001) respectively. One patient had *TP53* pathogenic variant which was detected in acfDNA but missed on tissue. Importantly in 5 patients for whom tumor pathogenic variant analysis was non-contributive, NGS on cftDNA from ascites was informative and uncovered one pathogenic variant of *BRCA2* with obvious immediate implications for germline testing and treatment.

However, there is limitation to this approach, mainly linked to the availability of ascites. Even though majority of patient with stade III/IV ovarian cancer has peritoneal fluid at diagnosis (98%), in some cases, patients requiring HRD testing may not exhibit peritoneal fluid or even peritoneal carcinomatosis and only have adenopathy making the analysis of ascites cfDNA unfeasible for HRD assessment. However, this represent a small proportion of patient with PARPi approved indication.

The determination of the HRD status is now mandatory for any high-grade ovarian cancer and can be obtained by CGP testing of *BRCA1* and BRCA*2* and genomic instability score. The two genomic instability scores used in registration clinical trials are the FoundationOne® cdx (Foundation Medicine®) and MyChoice® cdx (Myriad®) However, in our study, 25% of patient didn’t have an informative HRD status because of non-contributive NGS or Myriad® GIS on tumor samples. The use of acfDNA could be a useful and faster alternative to tumor DNA analysis for *BRCA* pathogenic variant analysis as well as genomic instability score with alternative techniques. Indeed, in all acfDNA samples in our study, we were able to measure genomic instability using sWGS thus providing a proof of principle. sWGS HRD is based on the number of large-scale genomic alteration (LGA) which is defined as intra-chromosome arm CNA breaks with adjacent segments ≥ 10 Mb. While this specific algorithm has not yet been approved for clinical use in ovarian cancer, HRD signatures on sWGS by different academic (SWGS v2) and commercial (SeqOne HRD score) algorithms are undergoing validation in clinical samples from patients in phase III trials of PARPi (PALOA-1) [19, 20]. Our results support that genomic instability testing is feasible on cftDNA from peritoneal fluid.

## Conclusion

Tumor genomic profiling on cftDNA from peritoneal fluids is feasible, yields high quality and quantity tumor DNA. Importantly this approach has a fast testing turn-around time and only requires 20 ml of ascites so that > 95% of patients with advanced OC would be eligible to this approach. This acftDNA is suitable for assessing HRD status combining both *BRCA1/2* pathogenic variant analysis and genomic instability score. This approach can even be of value in the rare event of absence of ascites by performing peritoneal washings. PARP inhibitors are bringing meaningful improvements in progression- free and overall survival to women with advanced ovarian cancer in case of *BRCA* or HRD status. Unfortunately, a significant proportion still ultimately relapse. Participation in clinical trials for relapsed disease almost invariably require archival tumor tissue. Any effort to save archival tumor tissue for later use in research trials or to guide biomarker driven treatment decisions in the future remains a priority.

### Supplementary Information

**Additional file 1: Supp data 1.** list of 65 genes included in the second CGP. **Supp data 2.** list of 109 genes included in the first CGP. **Supp data figure 1.** Correlation Plot of Variant Allele Frequencies (VAFs) of TP53 between Tissue and Ascites.

**Additional file 2**.

## Data Availability

The data that support the findings of this study are not openly available due to reasons of sensitivity and are available from the corresponding author upon reasonable request.
