# Supplementary material for: Homologous recombination deficiency (HRD) testing on cell-free tumor DNA from peritoneal fluid
Source: Mol Cancer. 2023 Nov 6;22:178. doi: 10.1186/s12943-023-01864-1 (PMC10626673; doi:10.1186/s12943-023-01864-1)
Supplement: Supplementary file 1 — Additional file 1: Supp data 1. list of 65 genes included in the second CGP. Supp data 2. list of 109 genes included in the first CGP. Supp data figure 1. Correlation Plot of Variant Allele Frequencies (VAFs) of TP53 between Tissue and Ascites. [file 12943_2023_1864_MOESM1_ESM.docx]

**SUPPLEMENTARY DATA :**

***Supp data 1. list of 65 gene included in the second CGP.***

**AKT1** (NM_005163.2), **ALK** (NM_004304.4), **APC** (NM_000038.5), **ARID1A** (NM_006015.4), **ATM** (NM_000051.3), **BAP1** (NM_004656.3), **BARD1** (NM_000465.3), **BRAF** (NM_001354609.1), **BRCA1** (NM_007294.3),**BRCA2** (NM_000059.3), **BRIP1**(NM_032043.2), **CCNE1**(NM_001238.3), **CDH1** (NM_004360.4), **CDK12**(NM_016507.3), **CHEK2**(NM_001005735.1), **CTNNB1**(NM_001904.3), **DICER1**(NM_030621.4), **EGFR**(NM_005228.4), **ERBB2**(NM_004448.3), **ESR1**(NM_000125.3), **EZH2**(NM_004456.4), **FANCA**(NM_000135.2), **FBXW7**(NM_033632.3), **FGFR1**(NM_023110.2), **FGFR2**(NM_000141.4), **FGFR3**(NM_000142.4), **FH**(NM_000143.3), **FOXL2**(NM_023067.3), **H3F3A**(NM_002107.4), **HIST1H3B**(NM_003537.3), **HRAS**(NM_005343.3), **IDH1**(NM_005896.3), **IDH2**(NM_002168.3), **KIT**(NM_000222.2), **KRAS**(NM_033360.3), **MAP2K1**(NM_002755.3), **MAP2K2**(NM_030662.3), **MET**(NM_001127500.2), **MLH1**(NM_000249.3), **MSH2**(NM_000251.2), **MSH6** (NM_000179.2), **MYOD1**(NM_002478.4), **NBN**(NM_002485.4), **NRAS**(NM_002524.4), **NTRK1**(NM_002529.3), **NTRK2**(NM_006180.4), **NTRK3**(NM_001012338.2), **PALB2**(NM_024675.3), **PDGFRA**(NM_006206.5), **PIK3CA**(NM_006218.2), **PMS2**(NM_000535.5), **POLD1**(NM_002691.3), **POLE**(NM_006231.3), **PTEN**(NM_000314.6), **RAD51**(NM_001164269.1), **RAD51B**(NM_001321821.1), **RAD51C**(NM_058216.2), **RAD51D**(NM_001142571.1), **RAD54L**(NM_001142548.1), **RET**(NM_020975.5), **RNF43** (NM_017763.4), **ROS1**(NM_002944.2), **SF3B1** (NM_012433.2), **STK11**(NM_000455.4), **TP53**(NM_000546.5)

***Supp data 2. list of 109 gene included in the first CGP.***

**AKT1** (NM_005163.2 ; exon 3), **AKT2** (NM_001626.6 ; exon 3), **AKT3**(NM_005465.7 ; exon 2), **ALK** (NM_004304.4 ; exons 20 to 29), **APC** (NM_000038.5 ; exon 2 to 16), **AR** (NM_000044.6 ; exon 4 to 7), **ARID1A** (NM_006015.4 ; exons 1 to 20), **ATM** (NM_000051.3; exons 2 to 63), **BAP1** (NM_004656.3 ; exons 1 to 17), **BARD1** (NM_000465.3 ; exons 1 to 11), **BRAF** (NM_004333.6 ; exons 11 et 15), **BRCA1** (NM_007294.3 ; exons 2 to 3 et 5 to 24), **BRCA2** (NM_000059.3 ; exons 2 to 27), **BRIP1**(NM_032043.2 ; exons 2 to 20), **CCND1** (NM_053056.2 ; exon 1 to 5), **CCNE1** (NM_001238.3 ; exons 2 to 12), **CDH1** (NM_004360.4 ; exons 1 to 16), **CDK12** (NM_016507.3 ; exons 1 to 14), **CDK4** (NM_000075.4 ; exon 2), **CDK6** (NM_001259.7 ; exon 2 to 8), **CDKN2A** (NM_000077.4_p16)( NM_058195.3_p14), **CHEK2** (NM_007194.3 ; exons 2 to 15), **CREBBP** (NM_004380.3 ; exon 1 to 31), **CTNNB1** (NM_001904.3 ; exon 3), **CYP2D6** (NM_000106.6 ; exon 1 to 9), **DDR2** (NM_006182.3 ; exon 17), **DICER1** (NM_177438.2 ; exons 2 to 27), **DPYD**(NM_000110.4 ; exon 1 to 23), **EGFR** (NM_005228.5 ; exons 2 to 24), **ELP1** (NM_003640.5 ; exon 2 to 37), **ERBB2** (NM_004448.4 ; exons 8, 17 to 25), **ERBB3** (NM_001982.4 ; exon 3, 6, 8 to 9 et 17 to 23), **ESR1** (NM_000125.3 ; exons 4 to 8), **EZH2** (NM_004456.4 ; exon 16), **FANCA** (NM_000135.2 ; exons 1 to 43), **FBXW7** (NM_033632.3 ; exons 2 to 12), **FGFR1** (NM_023110.3 ; exons 4, 7, 12, 14 et 15), **FGFR2** (NM_000141.4 ; exons 3,7 to 9, 12, 14 et 17), **FGFR3**(NM_000142.4 ; exons 7, 9, 12, 14, 16 et 18), **FH** (NM_000143.3 ; exons 1 to 10), **FLCN** (NM_144997.7 ; exon 4 to 14), **FOXA1** (NM_004496.5 ; exon 2), **FOXL2** (NM_023067.4 ; exon 1), **GATA3** (NM_001002295.2 ; exon 2 to 6), **GNA11** (NM_002067.5 ; exon 4 et 5), **GNAQ** (NM_002072.5 ; exon 2, 4 et 5), **GNAS** (NM_000516.6 ; exon 6 , 8 et 9), **H3F3A** (NM_002107.4 ; exon 2), **HIST1H3B** (NM_003537.3 ; exon 1), **HOXB13** (NM_006361.5 ; exon 1 et 2), **HRAS** (NM_005343.3 ; exon 2 to 4), **IDH1** (NM_005896.3 ; exon 4), **IDH2** (NM_002168.3 ; exon 4), **KEAP1** (NM_203500.2 ; exon 2 to 6), **KIT** (NM_000222.2 ; exons 8 to 21), **KRAS** (NM_033360.5 ; exons 2 to 4), **MAP2K1** (NM_002755.3 ; exons 2 to 7), **MAP2K2** (NM_030662.3 ; exons 2 to 7), **MED12** (NM_005120.3 ; exon 2), **MEN1** (NM_130804.2 ; exon 3 to 11), **MET** (NM_001127500.3 ; exons 1 to 21), **MLH1** (NM_000249.3 ; exons 1 to 19), MSH2 (NM_000251.2 ; exons 1 to 16), MSH6 (NM_000179.2 ; exons 1 to 10), **MUTYH** (NM_001048171.1 ; exon 1 to 16), MYCN (NM_005378.6 ; exon 2), **MYOD1** (NM_002478.4 ; exon 1), **NBN** (NM_002485.4 ; exon 1 to 16), **NF1** (NM_001042492.3 ; exon 1 to 58), **NFE2L2** (NM_006164.5 ; exon 2), NRAS (NM_002524.4 ; exon 2 to 4), **NTRK1** (NM_002529.3 ; exons 13 to 17), **NTRK2** (NM_006180.4 ; exons 17 to 21), **NTRK3** (NM_001012338.2 ; exon 15 to 20), **NUDT15** (NM_018283.4 ; exon 1 to 3), **PALB2** (NM_024675.3 ; exons 1 to 13), **PDGFRA** (NM_006206.5 ; exons 12 to 21), **PIK3CA** (NM_006218.2 ; exons 2 to 21), **PIK3R1** (NM_181523.3 ; exon 2 to 16), **PMS2** (NM_000535.6 ; exons 1 to 15), **POLD1** (NM_002691.3 ; exons 5 to 15 et 23 to 24), **POLE** (NM_006231.3 ; exons 9 to 28), **PPP2R1A** (NM_014225.5 ; exon 5 et 6), **PTEN** (NM_000314.8 ; exon 1 to 9), **RAC1** (NM_018890.4 ; exon 2), **RAD51** (NM_001164269.1 ; exon 2 to 10), **RAD51B** (NM_133509.3 ; exons 2 to 11), **RAD51C** (NM_058216.2 ; exon 1 to 9), **RAD51D** (NM_002878.3 ; exon 1 to 10), **RAD54L** (NM_001142548.1 ; exons 1 to 18), **RARA** (NM_000964.3 ; exon 5 to 9), **RB1** (NM_000321.2 ; exon 1 to 27), **RET** (NM_020975.6 ; exons 8 to 19), **RHOA** (NM_001664.4 ; exon 2), **RNF43** (NM_017763.5 ; exons 2 to 10 ), **ROS1** (NM_002944.2 ; exons 36 to 42 ), **SF3B1** (NM_012433.3 ; exons 13 to 20), **SMAD4** (NM_005359.6 ; exon 2 to 12), **SMARCA4** (NM_001128849.2 ; exon 2 to 36), **SMARCB1** (NM_001317946.2 ; exon 1 to 9), **SPOP** (NM_003563.3 ; exon 5 et 6), **STK11** (NM_000455.4 ; exon 1 to 9), **SUFU** (NM_016169.3 ; exon 1 to 12), **TERT** (NM_198253.3 ; promoteur) **TP53** (NM_000546.5 ; exon 1 to 11), **TPMT** (NM_000367.5 ; exon 2 to 9), **U2AF1** (NM_006758.3 ; exon 2), **UGT1A1** (NM_000463.3 ; exon 1 to 5), **VHL** (NM_000551.3 ; exon 1 to 3), **MSI** : 24 target.

***Supp data figure 1 : Correlation Plot of Variant Allele Frequencies (VAFs) of TP53 between Tissue and Ascites***
